# Relationship of Thyroid Function with Renal Hemodynamics and Cholesterol Metabolism in Proteinuric Kidney Disease: A Pilot Study

**DOI:** 10.3390/metabo14020111

**Published:** 2024-02-07

**Authors:** Yoshitaka Iwazu, Kazuhiko Kotani, Taro Sugase, Daisuke Nagata, Toshiyuki Yamada

**Affiliations:** 1 Department of Clinical Laboratory Medicine, Jichi Medical University, Shimotsuke 329-0498, Japan; kazukotani@jichi.ac.jp (K.K.); yamadanji@jichi.ac.jp (T.Y.); 2 Division of Anti-Ageing Medicine, Center for Molecular Medicine, Jichi Medical University, Shimotsuke 329-0498, Japan; 3 Department of Nephrology, Jichi Medical University, Shimotsuke 329-0498, Japan; nagatad@jichi.ac.jp; 4 Division of Community and Family Medicine, Center for Community Medicine, Jichi Medical University, Shimotsuke 329-0498, Japan; 5 Seiikai Medical Clinic Nasu, Otawara 324-0034, Japan; kuma.ni.tyui@gmail.com

**Keywords:** nephrotic syndrome, free triiodothyronine, free thyroxine, para-aminohippurate, filtration fraction, hypercholesterolemia

## Abstract

Nephrotic syndrome and hypothyroidism are respectively reported to influence renal hemodynamics and hypercholesterolemia. However, the relationship of proteinuria-associated thyroid function with renal hemodynamics and cholesterol metabolism has yet to be determined in a simultaneous analysis of thyroid, renal, and cholesterol variables. We investigated the hypothesis that the changes in thyroid hormones by proteinuria may contribute to changes in cholesterol metabolism and renal hemodynamics by proteinuria. Twenty-nine patients (17 men and 12 women) with proteinuric kidney disease (mean age 46 years) were enrolled in a pilot study. Data for serum free triiodothyronine (FT3), free thyroxine (FT4), total cholesterol, and filtration fraction (FF; assessed by para-aminohippuric acid clearance) were used in variable-adjusted correlation analyses. The patients had the following data (mean ± standard deviation): urinary protein 5.18 ± 3.28 g/day, FT3 2.18 ± 0.44 pg/mL, FT4 1.03 ± 0.26 ng/dL, FF 0.27 ± 0.07, and total cholesterol 327 ± 127 mg/dL. There was a significant positive correlation of FT3 with FF (β = 0.58, *p* = 0.01) and a significant inverse correlation of FT4 with total cholesterol (β = −0.40, *p* = 0.01). A positive correlation of FT3 with FF and an inverse correlation of FT4 with total cholesterol were demonstrated in patients with proteinuric kidney disease. The proteinuria-associated reduction in serum thyroid hormone levels was correlated with hypercholesterolemia and the reduced glomerular FF. Further studies of these relationships are required.

## 1. Introduction

Proteinuria causes urinary loss of intermediate-sized plasma proteins [1] and results in a profound alteration of the metabolism of many plasma proteins and protein-bound substances, as well as certain cellular and tissue proteins [2]. Intermediate-sized proteins, including albumin, transferrin, immunoglobulin (Ig) G, hormone-binding proteins, and low molecular weight inhibitors of the clotting cascade, are lost in the urine and their concentration in plasma reduced. Since the plasma concentration of several proteins lost in the urine but not secreted by the liver, such as erythropoietin (EPO) and IgG, is not defended by increased synthesis, urinary loss of these proteins may cause reduced immunity, anemia, and deficiency syndromes [1]. In fact, urinary losses of EPO have been shown to cause EPO-deficiency anemia and prevent the normal increase in plasma EPO level in response to anemia and hypoxia in nephrotic syndrome (NS). Subcutaneous administration of recombinant EPO has been successfully used in the management of EPO-deficiency anemia in NS [2]. Thus, proteinuria is associated with endocrinologic abnormalities in addition to edema formation, alterations in cholesterol metabolism, and hypercoagulability [3,4].

Several studies have reported a high incidence of overt hypothyroidism and subclinical hypothyroidism (SCH) in patients with chronic kidney disease (CKD) not requiring chronic dialysis [5,6,7,8,9]. Because the kidney plays an important role in the metabolism, degradation, and excretion of thyroid hormones, CKD affects the hypothalamus pituitary thyroid axis. CKD affects thyroid function in many ways, including low circulating thyroid hormone levels, altered peripheral hormone metabolism, insufficient binding to carrier proteins, reduced tissue thyroid hormone content, and altered iodine storage in the thyroid gland (Figure S1) [9,10]. Proteinuria is a key sign of CKD and a cause of urinary loss of binding proteins such as the thyroxine-binding globulin transthyretin [5,11]. This can result in reduced total thyroxine and triiodothyronine levels in the circulation, leading to a state of hypothyroidism (Figure S1) [11]. In adult patients with proteinuria, SCH occurs 6 times more frequently than controls [12]. Patients with mild to moderate CKD can have hypercholesterolemia and high low-density lipoprotein (LDL) cholesterol, which is especially observed in patients with nephrotic proteinuria [13,14]. Hypothyroidism causes dyslipidemia in as many as 90% of patients, manifested in most cases by increased total and LDL cholesterol levels [15]. In addition, thyroid replacement therapy in early type 2 diabetic nephropathy patients with SCH may decrease urinary albumin excretion rate, and total and LDL cholesterol [16]. While the mechanistic link between dyslipidemia associated with kidney disease and that associated with thyroid disease remains unclear, it may suggest that thyroid, kidney, and lipid metabolism are interconnected (Figure S1), i.e., there is a thyroid–kidney cholesterol axis.

NS exhibits massive proteinuria with a lower glomerular filtration rate (GFR), which leads to acute kidney injury (AKI) and cholesterol dysregulation [17,18]. We experienced an adult case of NS with progression of AKI and exacerbated hypothyroidism accompanied by hypotension [19]. NS has been suggested to involve hypercholesterolemia with an increased vascular disease risk [15,18], while hypothyroidism may modulate dyslipidemias such as an increase in total cholesterol level [15].

These findings are currently fragmentary information, while massive proteinuria and hypothyroidism appear to influence renal and cholesterol dysregulation. Moreover, initiation of thyroid replacement therapy in CKD patients with SCH may potentially improve kidney function and reduce mortality [20]. Pilot clinical trials in patients with advanced proteinuric CKD and SCH demonstrated that administering levothyroxine decreased proteinuria and improved eGFR [21]. Thus, under our hypothesis that a reduced thyroid function with urinary thyroid hormone loss may be involved in reduced GFR and hypercholesterolemia, we performed a pilot study with simultaneous measurement of thyroid function, renal hemodynamics, and cholesterol metabolism in patients with proteinuric kidney disease.

## 2. Materials and Methods

### 2.1. Study Design and Patient Population

This study was conducted in the nephrology department of Jichi Medical University in Japan. Approximately 100 patients are admitted annually for renal biopsy in our department. Para-aminohippurate (PAH) clearance was routinely examined when renal biopsy was performed until March 2010. This study adheres to the principles of the Declaration of Helsinki. The requirement for written informed consent was waived by the Jichi Medical University ethics committee/institutional review board on 18 December 2017, because of the retrospective study design. This study was approved by the Jichi Medical University Hospital Bioethics Committee for Clinical Research (approval number: 17-110).

### 2.2. Patient Selection

Twenty-nine patients (17 men, 12 women; age 46 ± 18 years) with proteinuric kidney disease were enrolled in this cross-sectional study between April 2002 and March 2010. Inclusion criteria were age ≥ 16 years, urinary protein (UP) ≥ 0.15 g/24 h, thyroid function measured, and renal biopsy for diagnosis of kidney disease. Subjects with a history of primary thyroid disease were excluded. The underlying causes of CKD were as follows: minimal change disease (MCD) in nine; membranous nephropathy in eight; nephrosclerosis in three; immunoglobulin A nephropathy, focal segmental glomerulosclerosis, and lupus nephritis in two patients each; and IgA vasculitis, membranoproliferative glomerulonephritis, and crescentic glomerulonephritis in one patient each.

### 2.3. Data Collection

Each patient had data for serum free triiodothyronine (FT3), free thyroxine (FT4), thyroid-stimulating hormone (TSH), total cholesterol, and triglycerides measured by the central laboratory of the Jichi Medical University Hospital. Total cholesterol and triglycerides were measured by automated enzymatic assays using a Hitachi 7700 biochemical analyzer (Hitachi Hi-Tech Co., Ltd., Tokyo, Japan). Serum TSH, FT3, and FT4 were measured by an immunoenzymatic assay using E-test TOSOH II (TSH), E-test TOSOH II (FT3), and E-test TOSOH II (FT4) enzyme immunoassay with the AIA-1800 machine (Tosoh, Tokyo, Japan), respectively. Thyroid dysfunction was considered if the thyroid hormones were outside the reference range: FT3 (2.11–3.51 pg/mL), FT4 (0.84–1.44 ng/dL), and TSH (0.45–3.33 µIU/mL). Euthyroidism, including euthyroid sick syndrome, was considered if TSH levels were within the reference range. Overt hypothyroidism was defined as TSH > 3.33 µIU/mL, FT3 < 2.11 pg/mL, and FT4 < 0.84 ng/dL. SCH was considered if TSH > 3.33 µIU/mL and FT3 and/or FT4 were within the reference range.

### 2.4. Selectivity Index

Serum and urinary (in 24 h urine samples) IgG and transferrin were measured by the central laboratory of the Jichi Medical University Hospital and by an external laboratory (SRL, Inc., a commercial laboratory in Tokyo, Japan), respectively. Serum and Urine IgG were determined by immunoturbidimetry using a Hitachi 7180 biochemical analyzer (Hitachi Hi-Tech Co., Ltd., Tokyo, Japan). Serum and urine transferrin was measured by turbidimetric immunoassay and latex agglutination immunoassay. The selectivity index was calculated according to the Cameron and Blandford method [22] using the formula: selectivity index = (urinary IgG/serum IgG) × (serum transferrin/urinary transferrin).

### 2.5. Calculation of Renal Physiology

Renal function was evaluated with the GFR, renal blood flow (RBF), and renal plasma flow (RPF), assessed by the clearance of creatinine and PAH (aminohippurate sodium, 10%, Daiichi Sankyo Company, Tokyo, Japan), which has the benefit of being able to measure the effective renal plasma flow. A 1.0% PAH solution was infused over 10 min in the morning after an overnight fast. For continuous infusion, 0.5% PAH was administered at 3 mL/min (target plasma concentration 2 mg/dL). Water diuresis was induced by an oral water intake of 500 mL at the beginning. This enabled the subjects to empty their bladders by spontaneous micturition every 60 min. After a 1 h equilibration time, urine samples were collected and, midway through the collection period, a blood sample was drawn. The filtration fraction (FF) was calculated as GFR/RPF. Hematocrit, serum, and urinary PAH and creatinine were measured by the central laboratory of the Jichi Medical University Hospital. Serum and urine creatinine was determined enzymatically using a Hitachi 7700 biochemical analyzer. PAH concentrations were measured photometrically, by means of the N-1 naphthylethylenediamine and the anthrone method using a Corning 258 spectrophotometer.

### 2.6. Data Analysis and Statistics

Data are expressed as the mean ± standard deviation. The Wilcoxon signed-rank test and Chi-square test for categorical variables were used for statistical comparisons. Correlations of FT3 and FT4 with FF and total cholesterol were examined using Pearson correlation and multiple linear regression analyses with adjustment for age and drugs affecting renal hemodynamics and cholesterol metabolism. All analyses were performed using JMP ver. 7.0.1 (SAS Institute Inc., Cary, NC, USA). *p* < 0.05 was considered significant.

## 3. Results

The characteristics of our cross-sectional study groups are summarized in Table 1. The patients (UP 5.18 ± 3.28 g/day) had FT3 2.18 ± 0.44 pg/mL, FT4 1.03 ± 0.26 ng/dL, TSH 7.90 ± 21.74 µIU/mL, FF 0.27 ± 0.07 (reference range 0.20–0.22), and total cholesterol 327 ± 127 mg/dL (reference range 120–220). FT3 and FT4 levels were relatively low, and TSH, FF, and total cholesterol were high. Sixteen patients (55%) showed thyroid dysfunction, which were 10 SCH and six overt hypothyroidisms. Three patients took renin–angiotensin system inhibitors and six were taking cholesterol-lowering agents with prednisolone.

**Figure 1:**
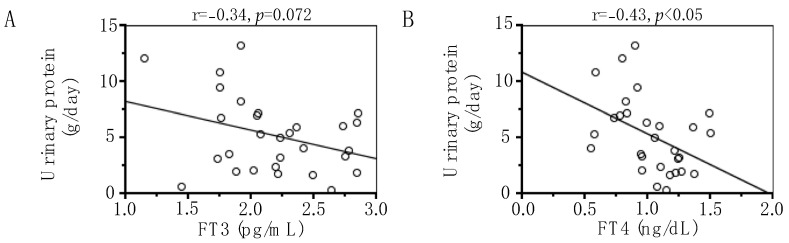
Correlations of serum free triiodothyronine (FT3) (**A**) and thyroxine (FT4) (**B**) concentrations with urinary protein excretion in the study population. The r value represents the nonparametric Spearman correlation coefficient.

Serum FT4 levels correlated negatively with UP (r = −0.43, *p* < 0.05) (Figure 1A), and serum FT3 levels tended to correlate negatively with UPE but did not reach statistical significance (r = −0.34, *p* = 0.072) (Figure 1B).

**Figure 2:**
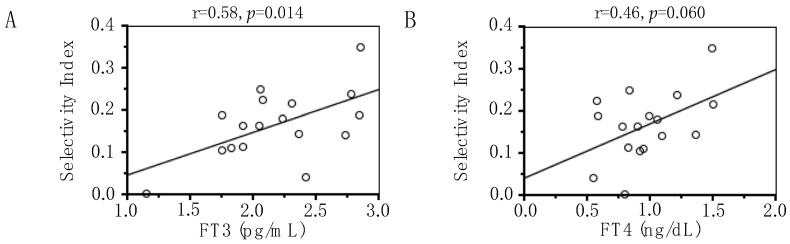
Correlations of serum free triiodothyronine (FT3) (**A**) and thyroxine (FT4) (**B**) concentrations with urinary protein excretion, selectivity index, and serum albumin levels in the UPE > 3.5 g/day group. The r value represents the nonparametric Spearman correlation coefficient.

Nephrotic-range proteinuria in the UP > 3.5 g/day group (*n* = 18) was associated with high UP (7.09 ± 2.67 vs. 2.05 ± 0.93 g/day), TSH levels (11.20 ± 27.05 vs. 2.50 ± 1.24 µIU/mL), the proportion of overt hypothyroid (33 vs. 0%) and low FT4 (0.95 ± 0.28 vs. 1.16 ± 0.13 ng/dL), albumin (2.2 ± 0.8 vs. 3.3 ± 0.6 g/dL) levels, and the proportion of euthyroidism (28 vs. 73%). No significant difference was found in FT3, CCr levels, and the proportion of subclinical hypothyroidism between the UP > 3.5 g/day group and the UP < 3.5 g/day group. The patients with nephrotic range proteinuria had selectivity index 0.17 ± 0.09. In the nephrotic-range proteinuria (UP > 3.5 g/day) group, serum FT3 levels correlated negatively with UP (r = −0.57, *p* < 0.05) and positively with selectivity index (r = 0.58, *p* < 0.05) (Figure 2A). Serum FT4 levels correlated positively with serum albumin levels (r = 0.48, *p* < 0.05), and tended to correlate positively with selectivity index, but did not reach statistical significance (r = 0.46, *p* = 0.060) (Figure 2B).

**Figure 3:**
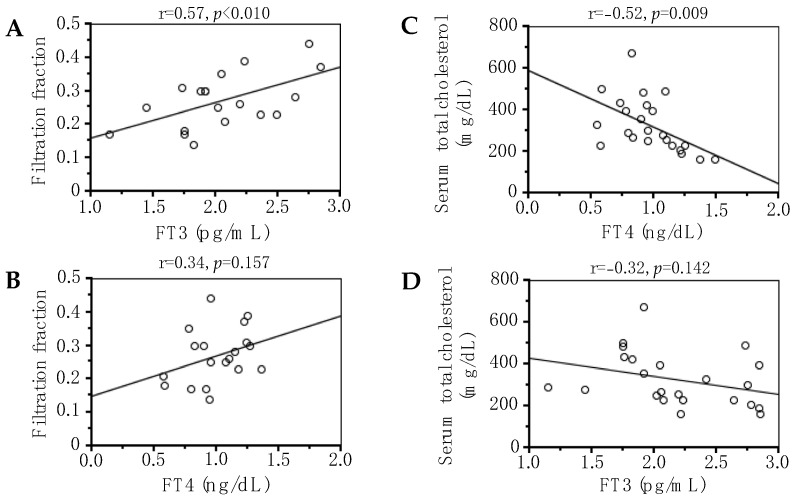
Correlations of serum free triiodothyronine (FT3) (**A**) and thyroxine (FT4) (**B**) concentrations with filtration fraction levels in the patients without renin–angiotensin–aldosterone system inhibitors. Correlations of thyroid function (**C**,**D**) with serum cholesterol levels in the patients without lipid-lowering agents with prednisolone. The r value represents the nonparametric Spearman correlation coefficient.

In the patients without renin–angiotensin–aldosterone system inhibitors, FF values were correlated positively with serum FT3 levels (r = −0.57, *p* < 0.05) (Figure 3A) but not serum FT4 levels (Figure 3B). In the patients without lipid-lowering agents with prednisolone, serum FT4 levels (r = −0.52, *p* < 0.01) (Figure 3C) but not serum FT3 levels (Figure 3D) were correlated negatively with total cholesterol levels. Both serum FT3 and FT4 levels were not correlated with serum triglyceride levels.

In all patients, as shown in Table 2, there was a significant positive correlation of FT3 with FF and a mild positive correlation of FT4 with FF. There was a mild inverse correlation of FF with total cholesterol (r = −0.27, *p* > 0.05). Multiple variable-adjusted analyses produced similar results for FT3 and FT4 with FF. There was also a significant inverse correlation of FT4 with total cholesterol and a mild inverse correlation of FT3 with total cholesterol. Multiple variable-adjusted analyses also produced similar results for FT3 and FT4 with total cholesterol. RPF and RBF values were not associated with serum FT3 and FT4 levels, although the RPF and RBF values were associated with age and CCr.

## 4. Discussion

This study is the first to show significant relationships of proteinuria-associated thyroid function with renal hemodynamics and cholesterol metabolism in proteinuric kidney disease. Albeit a pilot study, the simultaneous analysis of thyroid, renal, and cholesterol variables suggests that the changes in thyroid hormones by proteinuria may affect renal hemodynamics and cholesterol metabolism.

The positive correlation of serum FT3 with glomerular FF, reflecting glomerular transcapillary hydrostatic pressure, seems to be in line with earlier knowledge; hypothyroidism is reported to reduce glomerular transcapillary hydrostatic pressure with a single nephron GFR in thyroidectomized rats with remnant kidneys [23]. Because of a significant decrease in the glomerular FF for children with NS and AKI [17], in addition to a reversible reduction in GFR [24] and hypothyroidism [25,26] in patients with NS, a close interplay of low FT3 with low FF may underlie proteinuric kidney disease. The inverse correlation of serum FT4 with total cholesterol also seems to be consistent with earlier knowledge; hypercholesterolemia is known to be largely due to acquired LDL receptor deficiency in NS [18], while hypothyroidism reduces fractional clearance of LDL due to loss of hepatic LDL receptor density and activity [15] (Figure S1). Thus, the relationship of low FT4 with high total cholesterol may be caused by proteinuric kidney disease. Moreover, proteinuria [27] and hypothyroidism [28] have also been associated with increased cardiovascular events or mortality in patients with CKD not requiring chronic dialysis. It is suggested that hypothyroidism due to proteinuria may be partially involved in the cardiovascular and mortality risk of proteinuria.

The correlations of FT3 and FT4 with FF and total cholesterol showed similar directions, but the strength of correlations differed between FT3 and FT4 (Table S1). This might be due to the small number of subjects. The amount of daily urinary protein showed a negative correlation with serum FT4 and FT3 levels in children with NS [25]. We also demonstrated that increased proteinuria was associated with reduced serum FT4 levels, with a non-significant tendency toward reduced serum FT3 levels in adult proteinuric kidney disease patients. This finding may reflect the involvement of serum FT3 level not only in urinary loss but also in impaired peripheral conversion of T3. The increased urinary losses of protein-bound hormones or prohormones due to increased proteinuria alter their intravascular and extravascular distribution, their extrarenal clearance and the rate of their biosynthesis, the feedback regulation of the biosynthesis, and the release and peripheral conversion of such hormones [3,4]. However, no direct information is available on the effect of NS on the biosynthesis of either T4, T3, or their carrier proteins [3]. Likewise, data on the effect of NS on peripheral conversion of T4 to T3 and the integrity of the hypothalamic pituitary–thyroid axis are lacking [3]. Although, in adult NS, most patients remain euthyroid [10,29], in pediatric NS, almost half of all cases have abnormal thyroid hormone profiles [24], as in our study. This difference may be related to selectivity index, due to the higher proportion of MCD with low selectivity index in pediatric NS [24]. In this cross-sectional population study, selectivity index, which is based on the comparison of the clearance of IgG (molecular weight 170,000 Daltons), as a marker of high molecular weight proteins, and that of transferrin (molecular weight 80,000 Daltons), as a marker of intermediate-sized proteins [30], was low, and there was a significant association of reduced serum FT3 levels and high selectivity of proteinuria (low selectivity index) in patients with nephrotic-range proteinuria. Because nearly all circulating thyroid hormone is bound to three proteins, thyroxine-binding globulin, transthyretin or prealbumin, and albumin (molecular weight 50,000–70,000 Daltons) [31], highly selective proteinuria (low selectivity index) may result in more thyroid hormone-binding proteins being excreted in the urine with the same degree of proteinuria. Therefore, the impaired peripheral conversion of thyroid hormones and selectivity of proteinuria might both affect proteinuria-associated thyroid function. This clarification is an issue for further research.

This study has several limitations. In addition to the small sample size in this single-center study, the cross-sectional design incompletely determines causality, and generalizability of results could be limited. The participants are hospitalized for renal biopsy in a single university hospital, thus there may be a selection bias. Therefore, it may not represent the proteinuric kidney disease population. The data are based on Japanese patients with CKD; thus, it is not clear whether our data apply to other racial populations. Other variables associated with renal and lipid functions require examination in a multi-centric prospective observational study, and lifestyle factors may also be of importance.

## 5. Conclusions

A positive correlation of FT3 with FF and an inverse correlation of FT4 with total cholesterol were demonstrated in patients with proteinuric kidney disease. Further studies of these relationships are required to determine the interplay between these variables.

## Figures and Tables

**Table 1:** Characteristics of proteinuric kidney disease patients.

*n*	29
Age (years)	46.3 ± 17.5
Femal Sex (*n*)	12 (41%)
Serum total protein (g/dL)	5.3 ± 1.3
Serum albumin (g/dL)	2.6 ± 0.9
Urinary protein (g/day)	5.18 ± 3.28
Creatinine clearance (ml/min)	109.6 ± 44.0
Thyroid-stimulating hormone (µIU/mL)	7.90 ± 21.74
Serum free triiodothyronine (pg/mL)	2.18 ± 0.44
Serum free thyroxine (ng/dL)	1.03 ± 0.26
Filtration fraction	0.27 ± 0.07
Renal plasma flow (ml/min)	397 ± 128
Renal blood flow (ml/min)	658 ± 203
Serum total cholesterol (mg/dL)	327 ± 127
Serum triglycerides (mg/dL)	192 ± 196
Renin–angiotensin system inhibitor (*n*)	3 (10%)
Lipid-lowering agents and prednisolone (*n*)	6 (21%)
Euthyroid (*n*)	13 (45%)
Overt hypothyroid (*n*)	6 (21%)
Subclinical hypothyroid (*n*)	10 (34%)

**Table 2:** Correlations of FT3 and FT4 with filtration fraction and total cholesterol levels.

Variable (Units)	FT3		FT4	
	r (*p* Value)	β (*p* Value)	r (*p* Value)	β (*p* Value)
For filtration fraction				
Age (years)	−0.17 (0.38)	−0.17 (0.40)	−0.17 (0.39)	−0.12 (0.61)
Filtration fraction (%)	0.55 (<0.01)	0.58 (<0.01)	0.29 (0.19)	0.32 (0.16)
Drugs ^a^ (existing use)	0.02 (0.93)	0.11 (0.58)	0.13 (0.49)	0.22 (0.33)
For total cholesterol				
Age (years)	−0.17 (0.38)	−0.17 (0.30)	−0.17 (0.39)	−0.28 (0.08)
Total cholesterol (mg/dL)	−0.23 (0.22)	−0.24 (0.23)	−0.47 (0.01)	−0.40 (0.01)
Drugs ^b^ (existing use)	−0.01 (0.94)	−0.02 (0.94)	−0.47 (0.01)	0.47 (0.01)

FT3: free triiodothyronine, FT4: free thyroxine. ^a^ Renin–angiotensin system inhibitors. ^b^ Cholesterol-lowering agents and prednisolone. r, Pearson correlation coefficient; β, standardized regression coefficient.

## Data Availability

Data supporting the findings of the study are available from the corresponding author upon reasonable request, after approval from the Ethics Committee.

## Supplementary Materials

The following supporting information can be downloaded at: https://www.mdpi.com/article/10.3390/metabo14020111/s1, Figure S1. Factors contributing to hypothyroidism in chronic kidney disease, and interrelationship between proteinuric kidney disease, hypothyroidism, and lipids (thyroid kidney cholesterol axis). LDL: low-density lipoprotein. Table S1. Correlations of filtration fraction and total cholesterol levels with free triiodothyronine (FT3) and thyroxine (FT4).
