# Supplementary material for: Relationship of Thyroid Function with Renal Hemodynamics and Cholesterol Metabolism in Proteinuric Kidney Disease: A Pilot Study
Source: Metabolites. 2024 Feb 7;14(2):111. doi: 10.3390/metabo14020111 (PMC10892275; doi:10.3390/metabo14020111)
Supplement: Supplementary file 1 [file metabolites-14-00111-s001.zip › metabolites-2845400-supplementary.pdf]

Relationship of thyroid function with renal hemodynamics and cholesterol metabolism in proteinuric kidney disease: A pilot study

Yoshitaka Iwazu,[1,2,3] Kazuhiko Kotani,[1,4] Taro Sugase,[5] Daisuke Nagata,[3] Toshiyuki Yamada[1]

[1] Department of Clinical Laboratory Medicine, Jichi Medical University, Shimotsuke, Tochigi 329-0498, Japan

[2] Division of Anti-ageing Medicine, Center for Molecular Medicine, Jichi Medical University, Shimotsuke, Tochigi 329-0498, Japan

[3] Department of Nephrology, Jichi Medical University, Shimotsuke, Tochigi 329-0498, Japan

[4] Division of Community and Family Medicine, Center for Community Medicine, Jichi Medical University, Shimotsuke, Tochigi 329-0498, Japan

[5] Seiikai Medical Clinic Nasu, Otawara, Tochigi, 324-0063, Japan

Corresponding author

Yoshitaka Iwazu, MD, PhD

Department of Clinical Laboratory Medicine, Jichi Medical University, 3311-1 Yakushiji, Shimotsuke 329-0498, Tochigi, JAPAN

TEL: +81-285-58-7346, FAX: +81-285-44-4869

E-mail: iwazu@jichi.ac.jp

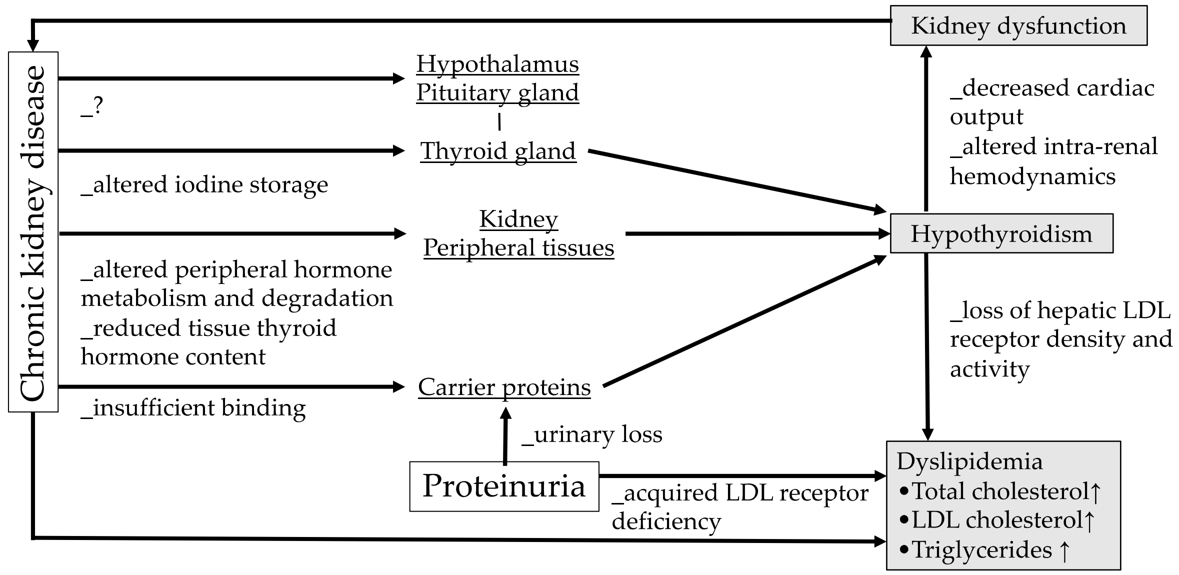

Figure S1. Factors contributing to hypothyroidism in chronic kidney disease, and interrelationship between proteinuric kidney disease, hypothyroidism, and lipids (thyroid kidney cholesterol axis). LDL: low-density lipoprotein.

Table S1. Correlations of filtration fraction and total cholesterol levels with free triiodothyronine (FT3) and thyroxine (FT4).

Variable (units)	Filtration fraction<br>β (P value)	Total cholesterol<br>β (P value)
FT3 (pg/mL)	0.51 (0.02)	-0.06 (0.76)
FT4 (ng/dL)	0.14 (0.48)	-0.45 (0.02)
